# High-throughput screening for modulators of *ACVR1* transcription: discovery of potential therapeutics for fibrodysplasia ossificans progressiva

**DOI:** 10.1242/dmm.023929

**Published:** 2016-06-01

**Authors:** Serena Cappato, Laura Tonachini, Francesca Giacopelli, Mario Tirone, Luis J. V. Galietta, Martina Sormani, Anna Giovenzana, Antonello E. Spinelli, Barbara Canciani, Silvia Brunelli, Roberto Ravazzolo, Renata Bocciardi

**Affiliations:** 1 Department of Neurosciences, Rehabilitation, Ophthalmology, Genetics, Maternal and Child Health and CEBR, Università degli Studi di Genova, Genova 16132, Italy; 2 Division of Immunology, Transplantation and Infectious Diseases, San Raffaele Scientific Institute, Milano 20132, Italy; 3 School of Medicine and Surgery, University of Milano-Bicocca, Monza 20900, Italy; 4 Medical Genetics Unit, IRCCS Istituto Giannina Gaslini, Genova 16147, Italy; 5 Medical Physics Department and Centre for Experimental Imaging, San Raffaele Scientific Institute, Milano 20132, Italy; 6 Dipartimento di Medicina Sperimentale, Università di Genova & IRCCS AOU San Martino-IST, Istituto Nazionale per la Ricerca sul Cancro, 16132 Genova, Italy

**Keywords:** *ACVR1*, Transcriptional regulation, BMP signaling pathway, FOP, Dipyridamole, High-throughput screening, Drug repositioning

## Abstract

The *ACVR1* gene encodes a type I receptor of bone morphogenetic proteins (BMPs). Activating mutations in *ACVR1* are responsible for fibrodysplasia ossificans progressiva (FOP), a rare disease characterized by congenital toe malformation and progressive heterotopic endochondral ossification leading to severe and cumulative disability. Until now, no therapy has been available to prevent soft-tissue swelling (flare-ups) that trigger the ossification process. With the aim of finding a new therapeutic strategy for FOP, we developed a high-throughput screening (HTS) assay to identify inhibitors of *ACVR1* gene expression among drugs already approved for the therapy of other diseases. The screening, based on an *ACVR1* promoter assay, was followed by an *in vitro* and *in vivo* test to validate and characterize candidate molecules. Among compounds that modulate the *ACVR1* promoter activity, we selected the one showing the highest inhibitory effect, dipyridamole, a drug that is currently used as a platelet anti-aggregant. The inhibitory effect was detectable on *ACVR1* gene expression, on the whole Smad-dependent BMP signaling pathway, and on chondrogenic and osteogenic differentiation processes by *in vitro* cellular assays. Moreover, dipyridamole reduced the process of heterotopic bone formation *in vivo*. Our drug repositioning strategy has led to the identification of dipyridamole as a possible therapeutic tool for the treatment of FOP. Furthermore, our study has also defined a pipeline of assays that will be useful for the evaluation of other pharmacological inhibitors of heterotopic ossification.

## Abstract (precis)

**Summary:** We describe the identification of dipyridamole as a potential therapeutic tool for FOP, through a series of *in vitro* and *in vivo* assays to screen and validate FDA-approved compounds.

## INTRODUCTION

Fibrodysplasia ossificans progressiva (FOP; OMIM 135100) is a rare genetic disease with a prevalence of about one per 2-million people. The inheritance is autosomal dominant, although most cases are due to sporadic new mutations (Shore et al., 2005). Individuals with FOP are characterized by a peculiar congenital toe malformation and, usually starting within the first decade of life, by a progressive heterotopic ossification (HO) that takes place following some types of injury (such as trauma, medical surgery, intramuscular immunization, infections) or spontaneously. Inflammatory soft-tissue swellings, commonly called flare-ups, progressively transform skeletal muscles, tendons, ligaments, fascia and aponeuroses into a second skeleton of heterotopic bone (Kaplan et al., 2008).

The FOP gene (*ACVR1*) encodes a type I receptor of bone morphogenetic proteins (BMPs), ACVR1 (also known as ALK-2). The most recurrent *ACVR1* mutation is in the glycine-serine (GS) domain (c.617G>A, p.R206H) (Shore et al., 2006). Additional mutations have been identified in the GS and in the kinase domain of the protein in 3% of all known individuals with FOP (for a review, see Kaplan et al., 2009; Bocciardi et al., 2009). The consequence of *ACVR1* mutations is an alteration of inter-intramolecular interaction of the mutant receptor that causes a deregulation of the downstream BMP signaling (Shore et al., 2006; Bocciardi et al., 2009; van Dinther et al., 2010; Song et al., 2010; Groppe et al., 2011; Chaikuad et al., 2012).

At present, no established medical treatment is available for FOP. Early diagnosis prevents unnecessary interventions, such as biopsies or surgical operations that can exacerbate the progression of the disease, and high-dose glucocorticoids are used in the management of inflammatory flare-ups (Kaplan et al., 2013).

In recent years, much effort has been devoted to designing new therapeutic approaches to FOP treatment and to identify new, potentially useful, drugs (Kaplan et al., 2013; Sanvitale et al., 2013; Yu et al., 2008a; Kitoh et al., 2013). A promising alternative to the discovery of new drugs is the drug repositioning strategy, in which a drug already developed for a specific disease can be used to treat a different condition. Drug repositioning reduces costs and accelerates the drug development process. Moreover, this approach might contribute to clarify the mechanism of action of a given compound by establishing a relationship between the molecular basis of the disease and the ability of the compound to intervene at a certain step of the disease process (Shameer et al., 2015).

A possible strategy to find drugs for the treatment of a genetic disease could rely on a sensitive, specific and fast cell-based assay. In this way, a large number of small molecules can be screened [high-throughput screening (HTS)] to find agents that correct the basic defect. The recent identification and characterization of the promoter region of *ACVR1* (Giacopelli et al., 2013) inspired us to develop an HTS assay by generating cells stably expressing the luciferase reporter gene controlled by a 2.9-kb region of the gene promoter. We expected that this type of assay would allow the identification of molecules that, by inhibiting the *ACVR1* promoter, would also negatively regulate the downstream signaling that is upregulated and hyper-responsive to BMPs because of the mutation in the receptor.

In this work, we describe the screening of a library of 1280 US Food and Drug Administration (FDA)-approved compounds, in order to identify modulators of *ACVR1* gene expression. Characterization of hit molecules included a series of second-level assays to evaluate the effect of compounds on chondrogenic and osteogenic differentiation models *in vitro* and *in vivo*.

We found that dipyridamole, commonly used as an antithrombotic and vasodilator drug, has an inhibitory effect on *ACVR1* expression, as well as on the whole BMP signaling pathway, and is able to affect chondrogenesis and osteogenesis, both in cellular assays and in a BMP-induced HO mouse model.

## RESULTS

### Screening of the Prestwick Chemical Library

Our primary screening was designed to find drugs that downregulate BMP signaling by targeting the expression of the *ACVR1* gene at the transcriptional level. Accordingly, we developed a quantitative assay based on expression of a reporter gene under the control of the *ACVR1* promoter. To this end, we generated clones of the ATDC5 cell line (mouse chondrogenic cell line derived from teratocarcinoma) stably expressing the luciferase coding sequence under the control of the 2.9-kb promoter of the gene, previously characterized by our group (Giacopelli et al., 2013). We obtained several clones that were expanded and selected for the level and stability of reporter gene expression over time. The availability of different clones, with putative different integration sites of the reporter construct in the genome of ATDC5 cells, allowed us to verify that the effect measured for a given compound was not related to a ‘position effect’ operated by the genomic region surrounding the reporter construct itself.

The generated cell system and the compound analysis procedure were tested by screening a small library of 43 molecules with chromatin-modifier properties. This allowed us to validate the protocol for the primary screening and provided us with a positive control because we identified resveratrol as a transcriptional activator of *ACVR1* gene expression (Fig. S1).

We used these cells to screen the Prestwick Chemical Library, which includes 1280 FDA-approved compounds, with the idea that ‘repositioning’ of an already approved drug could have the great advantage to overcome several steps of the drug discovery process. The screening detailed in Table 1 (see also Fig. S2) was performed in duplicate: compounds were added to cells seeded in 96-well plates for 24 h at the concentrations of 20 and 2 µM, respectively. We included in each plate DMSO, the vehicle in which compounds are dissolved, and resveratrol (20 µM) as a transcriptional activator of the *ACVR1* promoter and positive control. When we started this work, no transcriptional inhibitors of the *ACVR1* expression were known. However, during the screening of the second plate of the Prestwick Chemical Library, we detected dipyridamole as an inhibitor of *ACVR1* expression. Therefore, this compound was subsequently included in all the remaining plates as an additional control. To monitor the performance of the screening, we used the Z′-factor statistical parameter (Zhang et al., 1999). The calculated Z′-factor was 0.63±0.1 and 0.65±0.1 when considering resveratrol and dipyridamole, respectively. These values are considered optimal for an HTS assay (Zhang et al., 1999). During the primary screening, we also evaluated the toxicity of all tested compounds by an *in situ* fluorescence-based assay (Table 1 and see Materials and Methods for details). We therefore normalized the activity of the luciferase reporter gene driven by the *ACVR1* promoter with a fluorescence signal proportional to the number of viable cells at the end of the treatment. This allowed us to select molecules not affecting cell viability, inducing a reduction in the luciferase activity of at least 0.4- or an upregulation of at least 2.4-fold compared to cells treated with DMSO (Table S1).

**Table 1.**
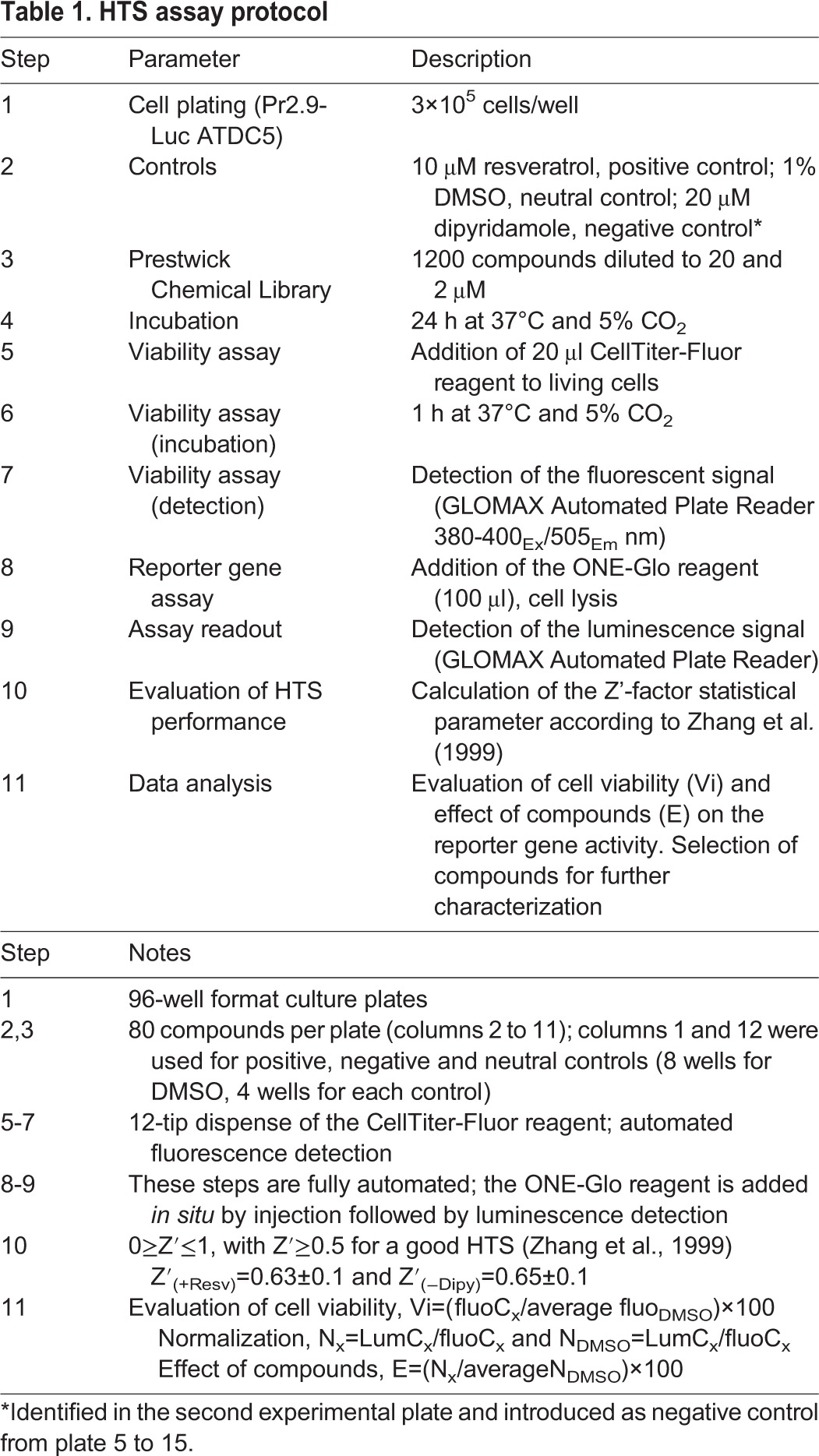
**HTS assay protocol**

### Validation assays of dipyridamole

According to our inclusion criteria, the primary screening provided a list of compounds putatively working as activators (4 hits) or inhibitors (18 hits) of *ACVR1* transcriptional activity (listed in Table S1). Among these latter molecules, we found that dipyridamole (abbreviated henceforth as Dipy), was the compound that, during the retesting of primary hits, generated the most reproducible and significant results. Therefore, Dipy was selected for further experimental confirmations.

**Fig. 1.**
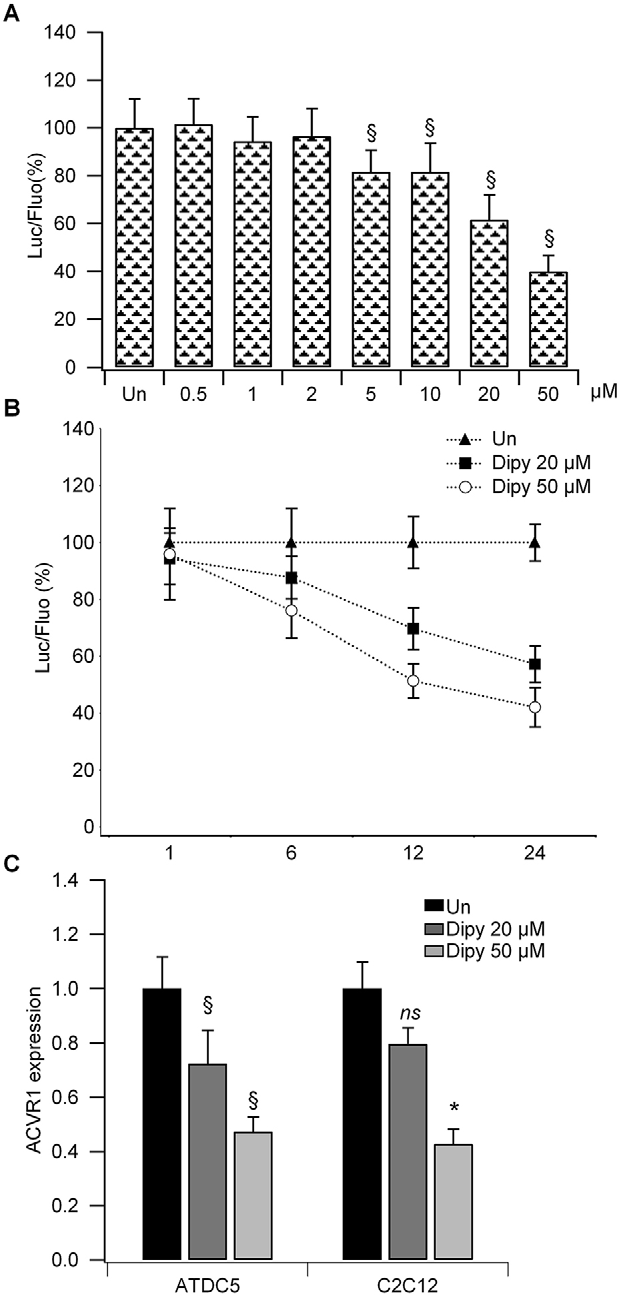
**Cellular assays of Dipy treatment.** (A) Dose-response curve of Dipy on the luciferase reporter gene controlled by the promoter region of *ACVR1* in ATDC5 cells (Pr2.9-Luc). The ratio of luciferase (Luc)/fluorescence (Fluo) was normalized to that obtained with DMSO [untreated (Un); value 100]. Bar graph represents the mean and s.d. of three independent experiments. ^§^*P*<0.001. (B) Time course of Dipy treatment in ATDC5 Pr2.9-Luc clones. The ratio of luciferase/fluorescence was normalized to that obtained with DMSO (Un; value 100) for each time point. (C) Effect of Dipy on the expression of *ACVR1* mRNA in native ATDC5 and C2C12 cells. Values were normalized on *GAPDH* and *β2M* and compared to expression level measured in cells treated with DMSO (Un). Bar graphs represent the mean and s.d. of at least three experiments, *P<0.01, ^§^*P*<0.001, ns, non-significant.

Dipy showed a dose-dependent suppression of the luciferase activity driven by the *ACVR1* promoter, with the strongest effect at 50 µM (Fig. 1A). The inhibition was detectable after 6 h of treatment for the highest dose, further increasing at 24 h (Fig. 1B). Normalization of the luciferase activity and monitoring of cell viability were obtained as described for the primary screening.

In accordance with the inhibitory effect of Dipy on the promoter of the *ACVR1* gene, we found that Dipy was able to downregulate the expression of *ACVR1* mRNA, as assessed by reverse-transcription quantitative PCR (RT-qPCR), both in native ATDC5 and C2C12 (mouse myoblast cell line) cells (Fig. 1C). After 24 h of treatment, we observed a gene-expression reduction of nearly 20% at 20 µM and 60% at 50 µM.

The effect of Dipy on the expression of genes encoding other type I and II receptors of the BMP family was also tested (Fig. S3). The highest degree of mRNA reduction was exerted on *ACVR1* (*Alk2*) but was also observed with *Alk3* and *BMPRII*. *Alk5*, involved in the growth differentiation factor (GDF)–transforming growth factor β (TGF-β) signaling cascade, and *Alk4*, *ActRIIa* and *ActRIIb* showed a low level of expression that was not affected by Dipy. Other type I receptors, such as *Alk1*, *Alk6* and *Alk7*, were not expressed in ATDC5 cells.

### Effect of dipyridamole on the Smad-dependent BMP pathway

**Fig. 2.**
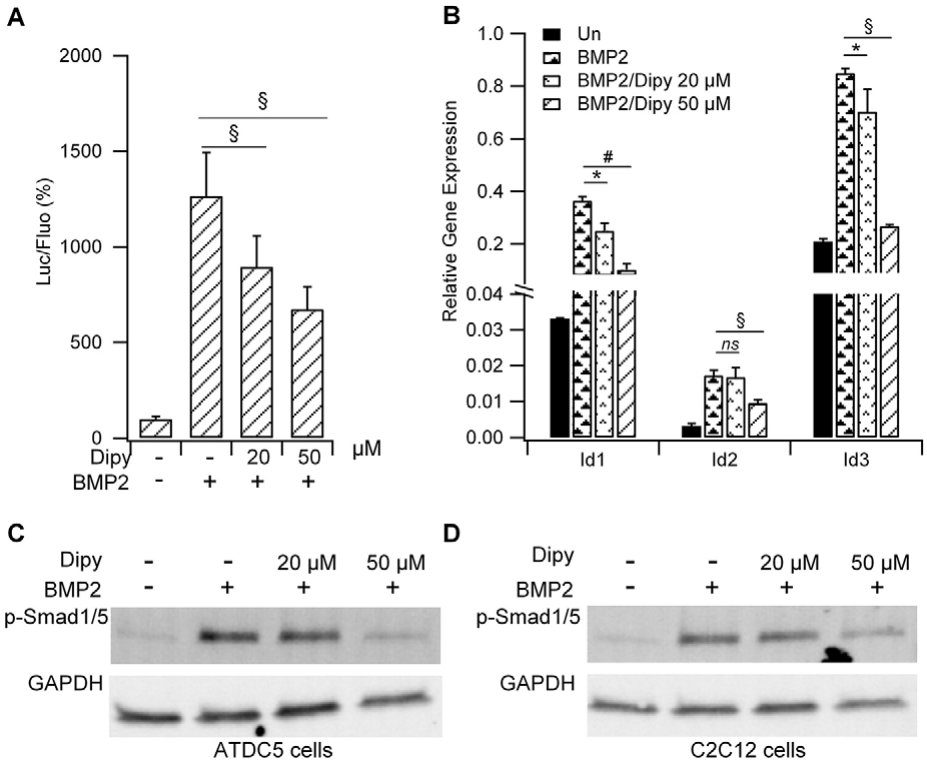
**Effect of Dipy on the BMP-mediated signaling pathway.** (A) Luciferase activity measured in ATDC5 BRE-Luc cells treated with the indicated doses of Dipy and activated with [50 ng/ml] BMP2. The ratio of luciferase (Luc)/fluorescence (Fluo) was normalized to that obtained with DMSO (value 100). Bar graph represents the mean and s.d. of three independent experiments. ^§^*P*<0.001. (B) Effect of Dipy on the expression level of *Id1*, *Id2* and *Id3* BMP target genes in native ATDC5 cells. Values were normalized on the *β2M* reference gene (relative quantification by the ΔC_t_ method: ratio reference/target=2^ΔCt^). Bars represent the mean and s.d. of three independent experiments. ns, non-significant; *P<0.05, ^#^*P*<0.01, ^§^*P*<0.001. (C,D) Effect of Dipy on the activation of the Smad-dependent pathway. ATDC5 (C) and C2C12 (D) cells were treated with Dipy and activated with [200 ng/ml] BMP2 for 1 h.

In order to test the effect of Dipy on the activation state of the Smad-dependent BMP signaling pathway, we generated ATDC5 clones stably expressing the luciferase reporter gene under the control of a minimal promoter carrying a BMP-responsive element (BRE-Luc) isolated from *Id1*, a well-known BMP target gene (Monteiro et al., 2008). Cells were treated with Dipy in the presence of BMP2 for 6 h. As reported in Fig. 2A, Dipy weakened the amplitude of the activation induced by BMP2 in a dose-dependent manner. Consistently, we found a downregulation in the mRNA expression of native *Id1*, *Id2* and *Id3* target genes, as assessed by RT-qPCR in ATDC5 cells (Fig. 2B), and a significant reduction in the phosphorylation state of the Smad1/5 proteins both in ATDC5 and C2C12 cells (Fig. 2C,D and Table S2 for immunoblot densitometric analysis).

### Effect of dipyridamole on chondrogenic differentiation

The heterotopic bone that forms in individuals with FOP derives from an endochondral ossification process. ATDC5 cells are able to differentiate into mature chondrocytes when grown in three-dimensional (3D) cultures in differentiating medium (Tare et al., 2005).

**Fig. 3.**
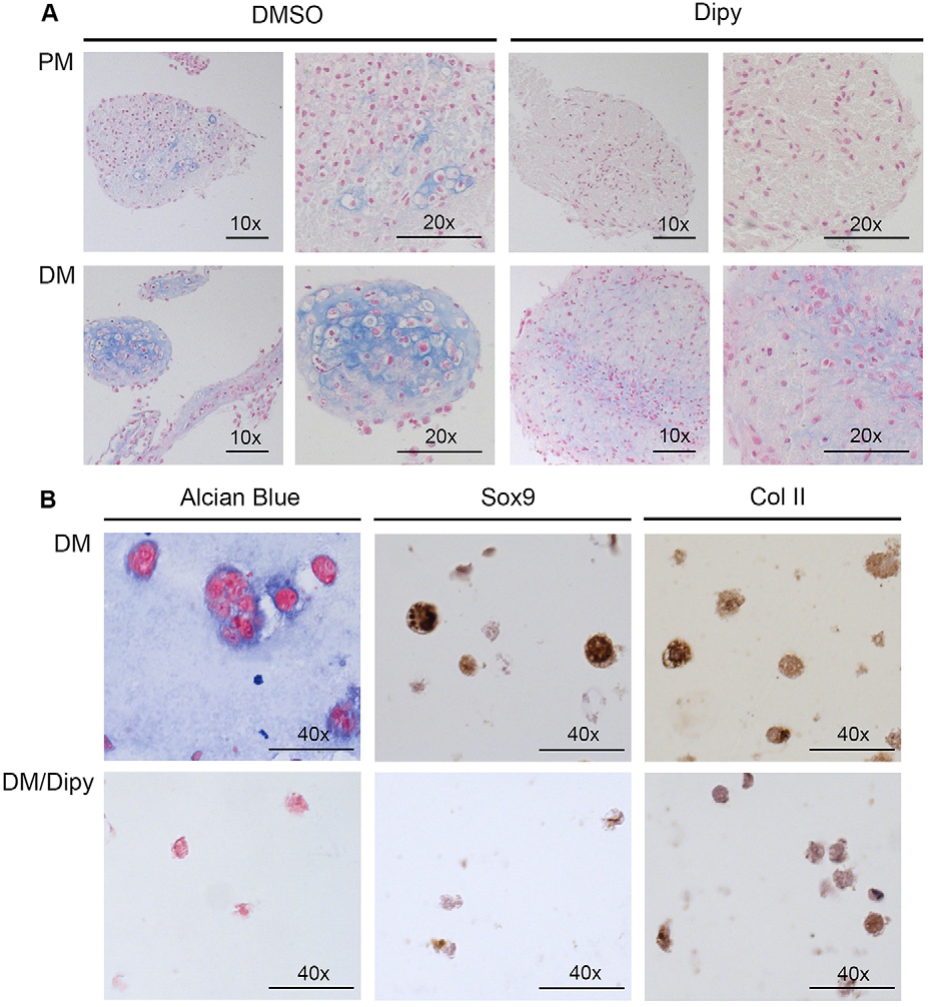
**Histological analysis of ATDC5 chondrogenic differentiation.** (A) Alcian Blue staining of sections from ATDC5 cell 3D cultures grown in proliferative medium (PM, upper panels) and in differentiation medium (DM, lower panels), in the presence of 50 µM Dipy or DMSO. Scale bars: 100 μm (10×) and 50 μm (20×). (B) Histological and immunohistochemical analysis of ATDC5 cells cultured as alginate spheres grown in DM in the presence of DMSO or 50 µM Dipy (upper and lower panels, respectively). Scale bars: 25 μm.

ATDC5 cells were induced to develop 3D pellets in the presence of differentiating medium (DM), with and without Dipy (50 µM). After 3 weeks of culture, pellets were embedded in paraffin, and histological sections stained with Alcian Blue to verify the deposition of glycosaminoglycans typical of the cartilage extracellular matrix. As shown in Fig. 3A (left panels), compared to what was observed in proliferative medium (PM), pellets grown in DM are characterized by the presence of cells with peculiar morphology, with typical lacunae embedded in the extracellular matrix. By contrast, pellets grown in the presence of Dipy, both in PM and DM, showed the presence of small and undifferentiated cells (Fig. 3A, right panels).

The result was confirmed in ATDC5 cells cultured in alginate spheres. In the presence of inductive medium, we observed changes in cell morphology correlating with the differentiation state (Fig. 3B, upper panels). By contrast, treatment with Dipy induced a significant reduction of extracellular-matrix deposition as assessed by Alcian Blue staining of sections (Fig. 3B, left panels) and reduced expression of matrix proteins Sox9 and collagen II (Col II) as assessed by immunohistochemical analysis with specific antibodies (Fig. 3B, central and right panels, respectively).

**Fig. 4.**
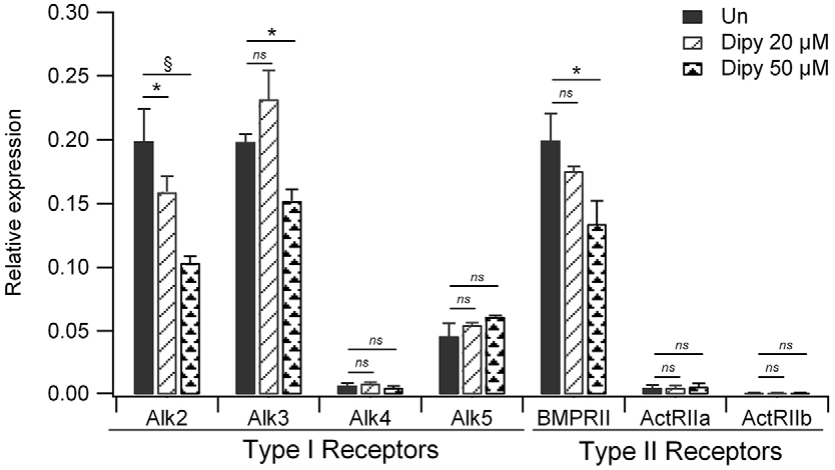
**Effect of Dipy on the expression of chondrogenic markers.** RT-qPCR on RNA extracted from ATDC5 cells cultured as alginate spheres for 14 days in differentiation medium. Bars show mean and s.d. of three independent experiments. Expression levels were normalized on *GAPDH* and *18S* and compared to that of cells at T_0_ (cells harvested at the beginning of the differentiation protocol). Un, untreated cells. ns, non-significant; *P<0.05, ^§^*P*<0.001.

In accordance, RT-qPCR analysis on mRNA extracted from cells cultured in alginate spheres showed that the expression level of *ACVR1* and markers of cartilage differentiation [*Runx2*, *Sox9*, *Col II* and collagen X (*Col X*)] was downregulated upon Dipy treatment compared to untreated cells (Fig. 4).

### Effect of dipyridamole on osteogenic differentiation

**Fig. 5.**
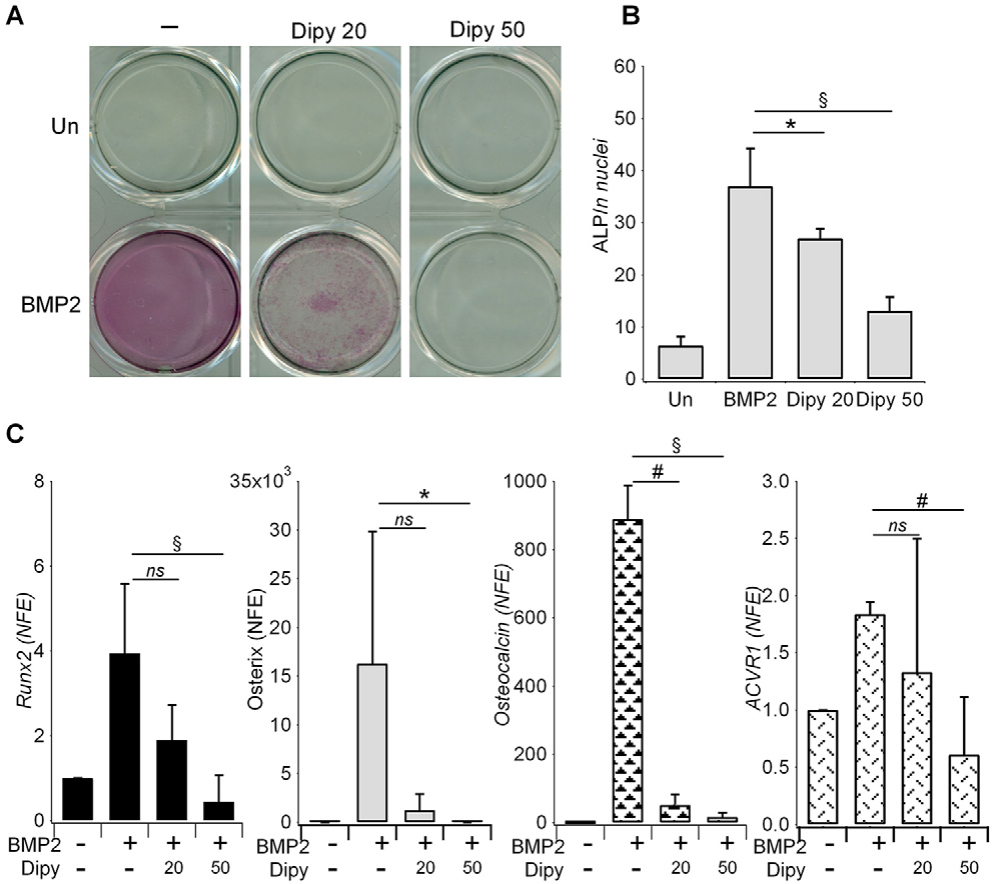
**Effect of Dipy on osteoblastic differentiation of C2C12 cells.** (A) Specific staining for alkaline phosphatase activity in C2C12 native cells cultured for 6 days in the presence of [200 ng/ml] BMP2, ±Dipy as indicated (20 or 50 μM). Un, untreated. (B) ALP activity measured in C2C12 cells treated as in A, normalized against the number of nuclei (cells) obtained by automated count after Hoechst staining in each well (see Materials and Methods and Fig. S3). (C) Gene expression level of osteogenic marker genes in C2C12 untreated or treated with Dipy (20 or 50 μM). Bar graphs represent mean and s.d. of three independent experiments. mRNA levels were normalized on *GAPDH* and *18S* and compared to that measured in untreated cells (value 1). NFE, normalized fold expression; ns, non-significant; *P<0.05, ^#^*P*<0.01, ^§^*P*<0.001.

We also investigated the effect of Dipy on the osteoblastic transformation of C2C12 cells upon BMP2 induction (Katagiri et al., 1994). As shown in Fig. 5A and B, Dipy caused a dose-dependent reduction in alkaline phosphatase activity without significantly affecting cell viability (Fig. S4). The effect was accompanied by a downregulation of the mRNA of markers typical of the osteoblastic differentiation – Runx2, osterix and osteocalcin – which was statistically significant at the highest dose (Fig. 5C). During the differentiation process, in the presence of Dipy, we confirmed the reduction in the expression of *ACVR1* mRNA.

### Effect of dipyridamole on heterotopic ossification in a BMP-induced mouse model

We examined the effect of Dipy on a BMP-induced model of HO *in vivo*. C57BL/6 2-month-old mice were injected with BMP2 intramuscularly in the quadriceps and treated with vehicle or 10 mg/kg (body weight) Dipy, administered daily by intra-peritoneal (IP) injection as described in Wang et al*.* (2013), according to two different experimental protocols as schematically represented in Fig. S5. Serum concentration of Dipy in mice was assessed according to Oshrine et al. (2005), and results were comparable to what was described in the same work (not shown).

**Fig. 6.**
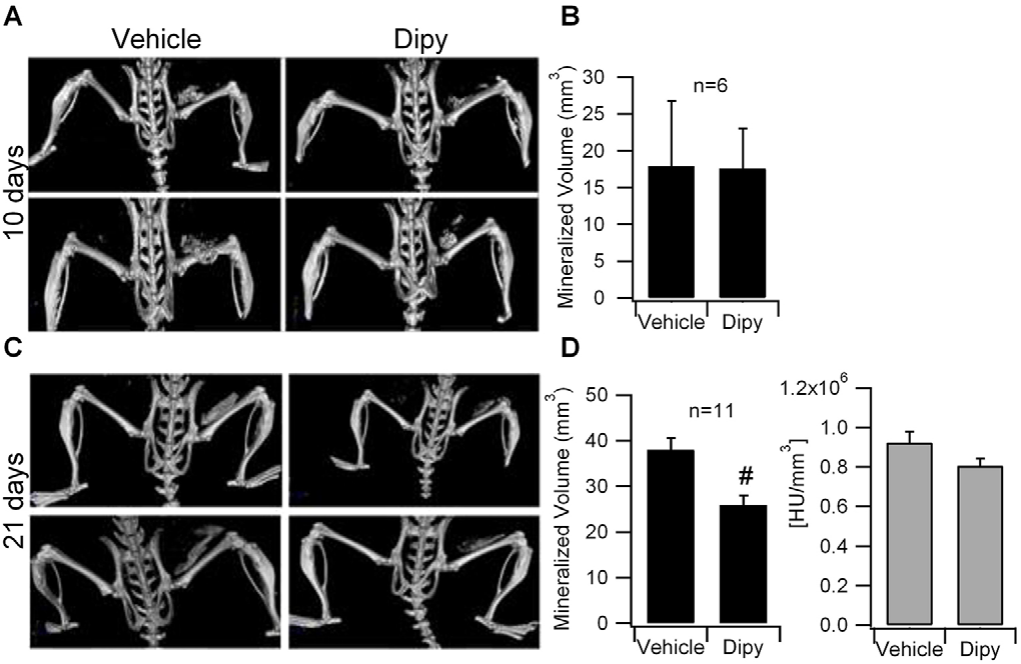
**Effect of Dipy on HO *in vivo*.** (A) Micro-computerized tomography (μCT) scans of C57BL/6 mice treated with vehicle or Dipy (10 mg/kg body weight) for 10 days. (B) Quantification of the mineralized ossicle volume. *n*=6 mice/condition. (C) μCT scans of C57BL/6 mice after 21 days of treatment with vehicle or Dipy (10 mg/kg body weight). (D) Quantification of the mineralized ossicle volume (left panel) and lesion bone density (HU/mm^3^, right panel). *n*=11 mice/condition. Bars represent mean and s.e.m. ^#^*P*<0.01. HU, Hounsfield unit.

**Fig. 7.**
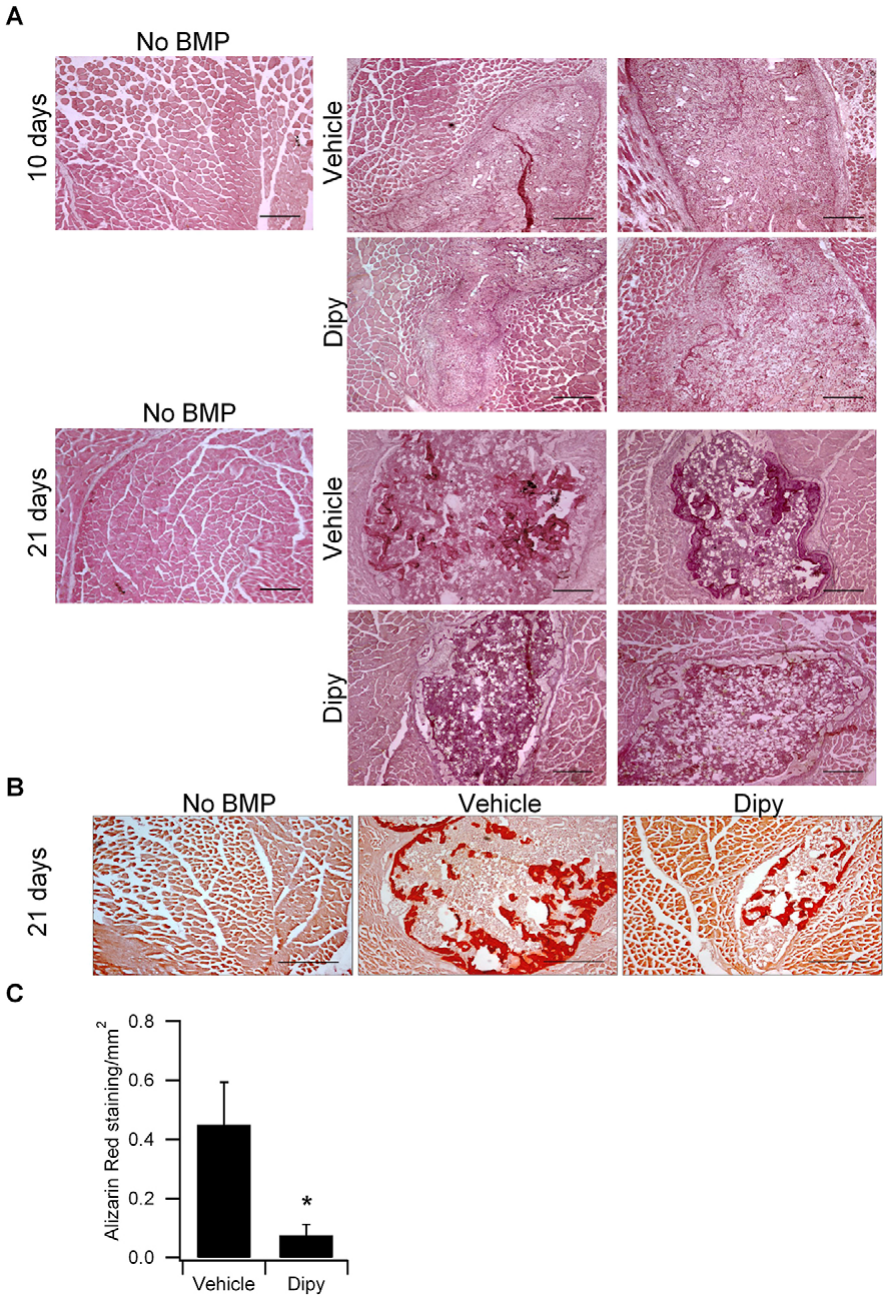
**Chronic Dipy treatment decreases BMP2-induced HO lesions and calcium deposition.** (A) Hematoxylin and eosin (H&E) staining showing lesions in muscle injected with BMP-2 and cardiotoxin in mice treated with vector or Dipy for 10 or 21 days. No lesions appear in muscle damaged without BMP. Scale bars: 1 mm. (B) Alizarin Red S staining showing calcium deposition in tissue sections of muscle injected with BMP2 and cardiotoxin in mice treated with the vehicle or Dipy for 21 days. Scale bars: 500 μm. (C) Graph representing the relative quantification of the Alizarin-Red-S-positive area. Data represent mean±s.e.m. *P≤0.05 (*n*=3, Dipy versus control).

Ossicle formation and HO volume were evaluated by μCT scan after 10 (*n*=6 for each group, Fig. S5 protocol A) and 21 (*n*=11 for each group, Fig. S5 protocol A) days of treatment. After 10 days of treatment, we observed highly variable volumes of HO (mineralized volume, mm^3^) in control mice and no significant difference in HO volume was observed in treated mice compared to controls (Fig. 6A,B). By contrast, after 21 days of treatment, μCT scans showed a significant reduction of HO volume in mice treated with Dipy compared to controls (Fig. 6C,D). Histological analysis revealed that HO lesions (Fig. 7A) in treated mice were reduced, possibly due to a delay in maturation. In particular, Toluidine Blue staining indicated a reduced deposition of cartilage matrix, also at 10 days of treatment (Fig. S6), whereas Alizarin Red staining at 21 days and quantification of the area of calcium deposition showed a decrease in lesions of Dipy-treated mice (Fig. 7B,C), in agreement with the μCT scan results.

Smad1/5 phosphorylation in the injured tissue was assessed at the two different time points, 10 and 21 days, by immunofluorescence with a specific anti-phospho-Smad1/5 (p-Smad1/5) antibody.

We observed that, at 10 days after injury, the number of cells showing Smad phosphorylation was higher than at 21 days. This is consistent with the ongoing osteogenic differentiation of the HO lesions at the early time point, when the extent of mature heterotopic ossification was still comparable in untreated and treated mice, as described above. This is also indicated by the shape and intensity of p-Smad staining per cell. Interestingly, the effect of Dipy at this stage was already detectable as a statistically significant reduction in the expression of p-Smad1/5 (Fig. S7). This decrease was still present as a trend at 21 days of treatment (Fig. S7B), when the overall number of p-Smad1/5-positive cells was reduced in the lesions of both control and treated mice.

When Dipy was administered to the mice starting from 10 days after the ossification trigger by BMP2 (Fig. S5, protocol B), the μCT-scan analysis of ectopic lesions (Fig. S8A) showed a trend of reduction (*P*=0.074) of the HO volume increase between 10 and 21 days (Fig. S8B,C). Histological analysis revealed that, at day 21, calcium deposition was also significantly reduced as assessed by Alizarin Red staining (Fig. S9A) and corresponding quantification (Fig. S9B).

## DISCUSSION

To date, no therapy is available to prevent or control HO in FOP patients. Therefore, intense work is being carried out to find potential therapeutic intervention essentially based on inhibition of BMP signaling using different approaches (Kaplan et al., 2013).

The rationale basis of a therapeutic approach for FOP is that small molecules might function as inhibitors, thus correcting the hyper-functioning BMP signaling pathway(s), either by inhibiting directly the receptor function or the transcriptional or post-transcriptional expression of the encoding gene, which will in turn result in the quantitative reduction of the receptor protein.

Following the identification of dorsomorphin as an inhibitor of BMP type I receptors, through HTS in zebrafish (Yu et al., 2008b), other inhibitors have been described (Yu et al., 2008a; Cuny et al., 2008; Hao et al., 2010). Previously published work demonstrated that treatment of bone-marrow-derived mesenchymal stem cells (MSCs) with RAR-γ agonists negatively regulates BMP signaling. This is due to the reduction of the intracellular concentration of p-Smads by a post-translational mechanism of degradation, supporting the idea that quantitative reduction of components of this pathway might cause a reduction of signaling function (Sanvitale et al., 2013; Shimono et al., 2011; Sheng et al., 2010).

In the current work, we introduced an HTS approach aimed at identifying potential therapeutic candidates acting by modulation of the *ACVR1* gene expression. The primary screening was made possible by the generation of a cell system consisting of murine ATDC5 cells stably expressing the luciferase gene controlled by the 2.9-kb promoter region of *ACVR1* that was previously identified and functionally characterized by our group (Giacopelli et al., 2013). Our method was able to pinpoint molecules with both positive and negative effects. However, in the context of FOP pathogenesis, in which activating mutations of *ACVR1* cause an inappropriate BMP-mediated signaling, our interest was focused on molecules able to reduce the expression level of the gene.

In addition to the primary screening, our approach included assays to confirm the effect of candidate molecules on the different steps of the BMP pathway, on chondrogenic and osteogenic differentiation processes and on HO *in vivo*. The experimental procedure described in this workflow can also be exploited to test compounds able to affect BMP signaling, even when discovered by other cell-based HTS assays or by *in silico* virtual screening approaches.

An advantageous approach to search for innovative treatments for rare disease in a relative short time is to perform an HTS approach with a drug repositioning purpose (Muthyala, 2011; Li and Jones, 2012; Sardana et al., 2011; Yamamoto et al., 2013). To this aim, we screened a library of 1280 FDA-approved compounds. We identified a list of interesting molecules with positive or negative effect and decided to focus on the candidate with the most significant effect as a transcriptional inhibitor, dipyridamole.

Validation assays confirmed a specific negative effect of Dipy on the *ACVR1* gene expression and demonstrated that such an effect resulted in the attenuation of the entire BMP-specific signaling pathway. This was demonstrated by the reduction of BMP2-induced activation tested by the luciferase reporter gene under the control of BMP-responsive element (BRE-Luc). Consistently, this effect was confirmed as reduced expression of the Smad-signaling target genes *Id1*, *Id2* and *Id3*, and as reduced phosphorylation of Smad1/5 mediators.

Although the highest effect of Dipy treatment was found to impact *ACVR1* (*Alk2*) expression, we found that Dipy could also affect the expression of other BMP receptors that can synergistically contribute to the downregulation of the overall BMP signaling, such as, among type I receptors, Alk3 or, among type II, BMPRII, which cooperates with ACVR1 as a type I partner in the receptor complex. It is of interest to note that Dipy did not affect the expression of specific receptors, such as *Alk5*, which is involved in cascade mediated by TGF-β type I, or *Alk4*, *ActRIIa* and *ActIIb*, which intervene in GDF-BMP signaling. This finding suggests that the downregulating effect of Dipy is mainly, but not exclusively, exerted on Alk2 (ACVR1), possibly because of common regulatory mechanisms for the expression of molecules belonging to the same family and participating in common pathways.

Because HO in FOP derives from an endochondral ossification process (Kaplan et al., 1993; Medici and Olsen, 2012; Shore, 2012), we set up assays to evaluate both chondrogenesis and osteogenesis. To simulate differentiation *in vitro*, we took advantage of the ability of ATDC5 cells to differentiate towards mature chondrocytes in 3D cultures, with cell morphological changes and deposition of glycosaminoglycans typical of the cartilage extracellular matrix. Using this assay, we observed that Dipy could inhibit chondrogenic differentiation.

C2C12 cells were used to evaluate osteogenic differentiation, which was inhibited by Dipy as indicated by the reduction in the alkaline phosphatase (ALP) activity and expression of different markers (Runx2, osterix and osteocalcin).

The effect of Dipy was also verified *in vivo* in a BMP-induced mouse model of HO (Medici et al., 2010). During the induction of the ectopic ossification process, triggered by the implantation of a BMP2-embedded Matrigel coupled to cardiotoxin (CTX) injection, muscle fibers degenerate and the site of injury is infiltrated by different populations of inflammatory cells that contribute to the orchestration of the subsequent repair/differentiation process (Zordan et al., 2014; Rigamonti et al., 2014). During the first week after injury, progenitor cells of different origin are then recruited to the site of the lesion (Bentzinger et al., 2013) and committed towards the endochondral ossification process by the local presence of BMP2. At 10 days, HO lesions are not completely differentiated, and recruitment and activation of cells is ongoing: this was consistent with our finding that, at this stage, the overall number of cells able to respond by activating a specific BMP2/Smad-dependent signaling was higher than what was observed at 21 days after BMP2 induction.

At the earliest time point, the effect of treatment with Dipy became evident as a statistically significant decrease in the number of cells expressing an activated BMP/Smad pathway inside the HO lesions that finally resulted in the reduction of the volume of mineralized heterotopic ossicles, of decreased deposition of extracellular matrix and of a reduced area of calcified nodules that we observed after 21 days of treatment.

Most interestingly, Dipy effect was evident also when mice were treated after the establishment of heterotopic ossification, and resulted in a reduced calcium deposition within the ectopic bone and a decreased mineralization. However, concerning the choice of an *in vivo* prevention strategy versus a treatment on established/ongoing lesions, it is important to consider that the course of FOP is episodic with quiescent phases, lasting even for years, and acute phases that can be triggered by several types of recognizable stimuli (trauma, vaccinations, infection, iatrogenic harms etc.), but that can also occur apparently spontaneously or, more likely, without a recognizable trigger. In this context, a treatment for FOP is ideally a drug that can be administered chronically or for long periods of time in order to prevent the occurrence of even unpredictable flare-ups, thus counteracting their consequences.

Dipy is a commercially available drug that was introduced on the market more than 50 years ago as a coronary vasodilator (Kadatz, 1959). At present, it is widely used as an antithrombotic and vasodilator agent both as monotherapy and in combination with aspirin to prevent secondary stroke or transient ischemic attack (Gresele et al., 2011; Balakumar et al., 2014; de Vos-Koppelaar et al., 2014). At the pharmacological level, Dipy acts by different mechanisms. By inhibiting the activity of phosphodiesterases 5 and 3 (PDE5 and PDE3), it increases the intracellular level of cyclic adenosine monophosphate (cAMP), which is a potent inhibitor of platelet activation, and of cyclic guanine monophosphate (cGMP), which has a vasodilator effect on smooth muscle, thus potentiating the platelet inhibitory actions of prostacyclin (PGI2) (Gresele et al., 2011; de Vos-Koppelaar et al., 2014; Kim and Liao, 2008; Yip and Benavente, 2011). Moreover, Dipy inhibits the re-uptake of adenosine by blocking the equilibrative nucleoside transporters (ENTs), thus increasing plasma levels of this nucleoside, which also plays a role in inhibiting platelet aggregation (Kim and Liao, 2008; Visser et al., 2005; Dresse et al., 1982; German et al., 1989), regulation of vascular tone, vasodilation, immunity and inflammation (Kim and Liao, 2008).

It should be noted that the effect of Dipy in our *in vitro* assay of C2C12 cells was not in accordance with other *in vitro* experiments performed to correlate adenosine level to osteoblast differentiation (Costa et al., 2011; He et al., 2013). This discrepancy might be due to differences in the cellular model and experimental conditions. Moreover, very recently, Mediero and colleagues (2015) reported that local daily injection of Dipy administered with a collagen sponge was able to induce bone regeneration and proposed this treatment as an alternative to recombinant human BMP2 (rhBMP2). At difference with the above report, in the BMP-induced *in vivo* model that we used, HO was locally triggered in quadriceps muscles, whereas treatment with Dipy was systemic, by daily IP injection. Moreover, it has been shown that, in humans, blood cells tend to accumulate the drug (Serebruany et al., 2009), and Dipy is able to inhibit at the mRNA level the production of TNF-α and MMP-9 of peripheral blood mononuclear cells (PBMCs) and derived macrophages (Massaro et al., 2013). Because several types of immune cells are recruited at the site of HO lesions, this might contribute to the overall effect observed in our HO model.

Several observations suggest a role of an immune-mediated response in FOP pathogenesis, in particular a possible relevant contribution to the episodic neo-formation of ectopic bone. In humans, FOP flare-ups can be triggered or exacerbated by trauma, immunizations, medical procedures and infections, all of which are conditions in which the immune response is solicited/stimulated (for a review, see Kaplan et al., 2016). Both in humans and in animal models, histological examination of early pre-osseous lesions has clearly demonstrated that several types of immune cells, such as lymphocytes, monocytes/macrophages and mast cells, are readily recruited to these sites (Kaplan et al., 1993; Chakkalakal et al., 2012). In addition, it has been demonstrated that *in vivo* targeted ablation of macrophages or of macrophages and mast cells concomitantly leads to a significant reduction in the ectopic bone formation in FOP mouse models (Kan et al., 2009; Convente et al., 2015).

The deregulated BMP signaling in cells harboring the mutated *ACVR1* gene might contribute to the amplification of inflammatory pathways (Convente et al., 2015); moreover, it has been recently demonstrated that the presence of FOP mutations specifically confers to the mutated receptor the ability to respond to activin A (Hatsell et al., 2015; Hino et al., 2015). Activin A is a ligand member of the TGF-β superfamily that is rapidly released during inflammation and considered a crucial mediator of inflammation and immunity. Its activity is stimulated by inflammatory cytokines, Toll-like receptor ligands and oxidative stress, and it is involved in regulating growth and maturation of mast cells, monocyte/macrophage differentiation, and interaction between natural killer (NK) and dendritic cells (Funaba et al., 2003; Ogawa and Funaba, 2011; Aleman-Muench and Soldevila, 2012; Seeger et al., 2014).

Given the known pleiotropic effect of Dipy, with anti-inflammatory, anti-oxidant and anti-proliferative properties, and the complexity of the action of a drug *in vivo*, related to its absorption, metabolism and distribution, we cannot exclude that the observed decrease of ectopic ossification in our *in vivo* model might depend on the involvement of different pathways. However, we showed that Dipy is also able to affect specifically the Smad-dependent pathway in HO lesions of treated mice.

Summarizing, the overall effect of Dipy on the process of HO *in vivo* might be mediated by different mechanisms of action, such as the metabolic effect of extracellular adenosine, regulatory properties on differentiation and activation of immune cells, and anti-inflammatory action.

In conclusion, our study implicates this molecule as a candidate drug for the treatment of FOP, considering the great advantage that Dipy is already widely used in the therapy of cardiovascular disorders, and that safety and adverse effect profiles have already been evaluated and established.

## MATERIALS AND METHODS

### Chemicals and reagents

The Prestwick Chemical Library was purchased from Prestwick Chemical (Illkirch-Graffenstaden, France) and supplied in a special academic format with the 1280 FDA-approved compounds at 10 mM concentration in DMSO, in 16 96-well format plates, each containing 80 compounds.

Resveratrol (CAS no. 501-36-0, Enzo Life Sciences, Farmingdale, NY, USA) and dipyridamole (CAS no. 58-32-2, Sigma, Buchs, SG, Switzerland) were dissolved in DMSO and prepared as 1 M and 200 mM stock solutions, respectively.

Recombinant human BMP2 [rhBMP2; Chinese hamster ovary (CHO)-derived, R&D Systems, Minneapolis, MN, USA] was prepared as a 100 µg/ml stock solution in 4 mM HCl containing 0.1% bovine serum albumin (BSA; Sigma-Aldrich, Buchs, SG, Switzerland).

Antibodies for western blot analyses were: anti-p-Smad1/5 (13820S, Cell Signaling, Danvers, MA, USA), anti-GAPDH (MAB3749, Millipore, Billerica, MA, USA), and horseradish peroxidase (HRP)-conjugated anti-rabbit and anti-mouse secondary antibodies (Dako, Glostrup, Denmark). For immunohistochemical analyses, the following antibodies were used: rabbit anti-bovine polyclonal antibody anti-collagen type II (AB746P, Millipore, Billerica, MA, USA), rabbit polyclonal antibody anti-Sox9 (AB5535, Millipore, Billerica, MA, USA), and anti-rabbit (K4002) and anti-mouse (K4000) EnVision System-HRP Labelled Polymer (Dako, Glostrup, Denmark).

### Expression plasmid preparation

The isolation of the genomic region, corresponding to the *ACVR1* promoter, was previously described by our group (Giacopelli et al., 2013). The whole 2.9-kb genomic fragment was subcloned in the pGL4.17 vector (Promega Corporation, Madison, WI, USA) upstream of the luciferase coding sequence as a reporter gene; this expression plasmid carries the Neomycin-resistance gene for selection of stable transfectants. The obtained reporter construct is reported as Pr2.9-Luc throughout the present work. A second reporter gene construct was also prepared by isolating a minimal promoter containing the BMP-responsive element (BRE) of the *Id1* gene from the pGL3-(BRE)_2_Luc plasmid (kindly provided by Dr Peter ten Djike, Leiden University Medical Center, Leiden, The Netherlands) (Monteiro et al., 2008). BRE was transferred in to the pGL4.17 vector upstream of the luciferase reporter gene (referred to as BRE-Luc) plasmid, carrying the Neomycin-resistance gene that allowed the generation of stable transfectants as described below.

### Cell culture

ATDC5 cells (mouse chondrogenic cell line derived from teratocarcinoma) were obtained from the Cell Bank of the Riken BioResource Center upon material transfer agreement (MTA); C2C12 myoblasts were purchased from the ATCC Cell Biology Collection (LGC standards, Bury, Lancashire, UK). ATDC5 cells were routinely cultured in complete medium consisting of 1:1 mixture of Dulbecco's modified Eagle's medium and Ham's F-12 medium (DMEM/F12), containing 5% fetal bovine serum (FBS, Gibco, Thermo Fisher Scientifics, Waltham, MA, USA). C2C12 cells were cultured in DMEM containing 10% FBS. Both culture media were supplemented with 2 mM glutamine, 100 U/ml penicillin, 0.1 mg/ml streptomycin (EuroClone^®^ S.p.a., Pero, MI, Italy), and cells were maintained at 37°C in a humidified atmosphere with 5% CO_2_. Where indicated, in depletion media, FBS was replaced by 0.1% BSA (Sigma-Aldrich, Buchs, SG, Switzerland).

### Transfection and generation of the cellular system

For stable transfection, ATDC5 cells were plated in 100-mm dishes at a density of 2×10^4^/cm^2^. The next day, cells were transfected with 30 µg of the Pr2.9-Luc and of the BRE-Luc constructs, using the Lipofectamine 2000 reagent protocol (Invitrogen, Thermo Fisher Scientifics, Waltham, MA, USA). After 24 h and for 2 weeks, transfected cells were maintained in complete medium containing 400 µg/ml of Neomycin/G418 (Sigma-Aldrich, Buchs, SG, Switzerland) as selective agent. Thereafter, Neomycin-resistant clones were picked up and expanded. For each clone, 1×10^5^ cells were collected after every cell-culture passage and lysed to evaluate the Luciferase activity with the ONE-Glo™ Luciferase Reporter Assay (Promega Corporation, Madison, WI, USA) according to the manufacturer's instruction. Clones showing stable expression of the reporter gene over the time course were considered suitable for our purposes and used to set up the culture and treatment protocols in 96-well format plates.

### Screening of the Prestwick Chemical Library in ATDC5 cells

A selected clone of ATDC5 Pr2.9-Luc was seeded into 96-well plates in depletion medium (3×10^5^ cells/well). After overnight culture, cells were treated with compounds at the final concentration of 20 μM and 2 μM. We tested 80 molecules in each plate; cells in columns 1 and 12 were treated with 1% DMSO as neutral control, resveratrol (10 µM) as positive control and dipyridamole (20 µM) as negative control (8 wells for DMSO, four wells for each control).

After 24 h, we measured the effect of the compounds on both cell viability and Luciferase activity by using the ONE-Glo™+Tox Luciferase Reporter and Cell Viability Assay (Promega Corporation, Madison, WI, USA) as suggested by the manufacturer. In brief, 20 µl of the CellTiter-Fluor Reagent were added *in situ* to living cells; after 1 h at 37°C, a fluorescent signal proportional to the number of viable cells in the culture well was measured by Glomax Multi Detection System (Promega Corporation, Madison, WI, USA). 100 µl of the second ONE-Glo Reagent were then added directly to each well to allow cell lysis and detection of the luciferase signal (Glomax Multi Detection System, Promega Corporation, Madison, WI, USA).

Fluorescence (fluo) and luminescence (*ACVR1* promoter activity; Lum) raw data were handled with the Instinct Software (Promega Corporation, Madison, WI, USA) and analyzed as an Excel spreadsheet. Cell viability (Vi) was first evaluated by comparing the fluo signal obtained in cells treated with compounds (fluoC_x_) versus that of cells exposed to the vehicle [Vi=(fluoC_x_/average fluo_DMSO_)×100, with 0≥Vi≥100]. In parallel, the effect of compounds (E) on *ACVR1* transcriptional activity was evaluated as follows: first, by normalizing luminescence signal over the fluorescence signal for each test well (N_x_=LumC_x_/fluoC_x_) and for the neutral control (N_DMSO_=Lum_DMSO_/fluo_DMSO_), then by comparing the normalized values of compounds with that of the vehicle [E=(N_x_/averageN_DMSO_)×100].

### RNA extraction and quantitative RT-PCR (RT-qPCR)

For expression studies, treated and untreated cells (ATDC5 and C2C12) were harvested and total RNA was isolated by using the RNeasy Mini Kit (Qiagen, Valencia, CA, USA), according to the provided protocol.

RNA was quantified with a Nanodrop Spectrophotometer (Thermo Scientific, Thermo Fisher Scientifics, Waltham, MA, USA), and first-strand cDNA was synthesized by the Advantage RT-for-PCR Kit (Becton) from 200 ng of total RNA.

Expression of endogenous *ACVR1* gene and of selected markers was evaluated through RT-qPCR using specific TaqMan Gene Expression Assay (Life Technologies, Thermo Fisher Scientifics, Waltham, MA, USA) (see Table S3 for specification). Samples were measured in triplicate and the results were normalized on reference genes *18S*, *GAPDH* and β2-microglobulin (*β2M*), depending on the cell line. qPCR was run on the IQ5 instrument from Bio-Rad and data analysis was performed using the provided Bio-Rad iQ5 software for Gene Expression Study.

### Western blot

For detection of p-Smad, 1.2×10^6^ cells were plated in 100-mm dishes in 1:1 complete/depletion medium for ATDC5 cells, and in depletion medium for C2C12 (DMEM containing 1% FBS). The next day, serum-starved cells were treated with Dipy for 24 h and, where indicated, with BMP2 (R&D Systems, Minneapolis, MN, USA) 200 ng/ml for 1 h. Cells were then washed once with PBS and lysed in 1× RIPA buffer (50 mM Tris HCl pH 7.5, 150 mM NaCl, 1% Nonidet P-40, 1% sodium deoxycholic, 0.1% SDS) containing phosphatase and protease inhibitors (PhosSTOP cocktail and Complete tablets, Roche, Basel, Switzerland). Protein concentration was determined by the Pierce™ BCA Protein Assay Kit (Thermo Scientific, Thermo Fisher Scientifics, Waltham, MA, USA) according to the manufacturer's protocol and 15 µg of total lysates run onto precasted 4-15% Mini Protean^®^TGX-gels (Bio-Rad, Hercules, CA, USA). Proteins were transferred onto PVDF membrane (Millipore, Billerica, MA, USA) and probed with the indicated primary antibody at 4°C overnight. After incubation with HRP-conjugated secondary antibodies, protein bands were revealed by chemiluminescence with the ECL kit (Pierce, Thermo Fisher Scientifics, Waltham, MA, USA) and detected with the ChemiDoc instrument (Bio-Rad, Hercules, CA, USA). Densitometric analysis of western blot signals was performed by using the ImageJ software.

### Culture in 3D pellets

ATDC5 cells were trypsinized from monolayer cultures and 1 ml of cell suspension with 5×10^5^ cells in DMEM was added to 15 ml polycarbonate sterile tubes according to Tare et al. (2005). The cell suspension was centrifuged at 400 ***g*** for 10 min at 4°C to obtain pellets that were cultured both in standard complete medium and in chondrogenic medium containing 10 ng/ml TGF-β3 (Calbiochem, Millipore, Billerica, MA, USA), 10^−8^ M dexamethasone (Sigma-Aldrich, Buchs, SG, Switzerland), 100 mM ascorbate-2-phosphate (Sigma-Aldrich, Buchs, SG, Switzerland), 1× insulin-trasferrin-selenium (ITS) solution (Life Technologies, Thermo Fisher Scientifics, Waltham, MA, USA). Pellets were cultured for 21 days in a humidified incubator at 37°C and 5% CO_2_. Pellets were swirled within to allow medium access to all sides and prevent adhesion to the inner walls of the tube. Once compact pellets were formed, both proliferative and differentiating media were replaced every 3 days and thereafter over the culture period. Three pellets from each group were harvested and processed for histological analysis.

### Culture in alginate spheres

ATDC5 cells were cultured in alginate spheres according to Culbert et al. (2014). Briefly, cell suspensions at 6.7×10^6^ cells/ml in 1.2% alginate acid sodium salt (Sigma-Aldrich, Buchs, SG, Switzerland) solution were extruded through 16-gauge needles as ∼30 µl drops in 30 ml of 102 mM CaCl_2_ (Sigma-Aldrich, Buchs, SG, Switzerland) in order to allow sphere formation. After drop solidification, cells/alginate spheres were washed with PBS and cultured in chondrogenic medium, replenishing every 3 days. A number of alginate spheres for each condition were formalin-fixed and processed for histological stainings and immunohistochemical assays. In parallel, spheres were also incubated with 55 mM sodium citrate (Sigma, Buchs, SG, Switzerland) to recover cells for total RNA extraction and expression analysis of markers specific for chondrogenesis such as *Runx2*, *Sox9*, *Col II*, *Col X*, aggrecan and also *ACVR1* by RT-qPCR with TaqMan Assays probes (Life Technologies, Thermo Fisher Scientifics, Waltham, MA, USA) (see Table S3 for specification).

### Histological analysis

Cell aggregates were fixed with 4% formaldehyde (Santa Cruz Biotechnology, Dallas, TX, USA) in PBS for 10-15 min, and embedded in paraffin according to standard protocols. Paraffin sections (5 μm) were obtained by microtome, dewaxed and rehydrated with decreasing ethanol solutions. For histological analysis, sections were stained with Alcian Blue 8GX (Sigma-Aldrich, Buchs, SG, Switzerland) following established procedures and viewed in transmitted and polarized light microscopy.

### Immunohistochemistry

Dewaxed and rehydrated sections were incubated with 3% hydrogen peroxide in methanol for 30 min to inhibit endogenous peroxidase activity, rinsed in PBS/0.2% Triton X-100, then were subjected to digestion with 1 mg/ml hyaluronidase in PBS, pH 6.0 for 15 min at 37°C prior to use. Sections were exposed to normal goat serum (Dako, Glostrup, Denmark) 1 h before incubation with the primary antibodies (24 h, 4°C). Slides were then washed with PBS (four times for 5 min each) and incubated with the HRP-conjugated secondary antibodies for 1 h at room temperature (RT). The peroxidase reaction was developed using 3,3′-diaminobenzidine tetrahydrochloride (DAB) as chromogens. After rinsing in distilled water, sections were dehydrated in increasing ethanol solutions, cleared in xylene and mounted.

### C2C12 cell culture and osteogenic differentiation

In order to induce C2C12 differentiation towards the osteoblastic lineage (Katagiri et al., 1994), 1.2×10^4^ cells were seeded in 6-well plates and cultured in complete medium supplemented with 5% FBS (low-mitogen medium). The day after, cells were treated with 300 ng/ml BMP2 (R&D Systems, Minneapolis, MN, USA) for 6 days. Where indicated, Dipy (50 µM) was also added to both standard and differentiating media.

Cells were processed to evaluate the alkaline phosphatase (ALP) enzymatic activity by the Alkaline Phosphatase (Sigma Diagnostics, Buchs, SG, Switzerland) kit following the manufacturer's instructions and total RNA was extracted to evaluate the expression of *Runx2*, osterix and osteocalcin by RT-qPCR with TaqMan Assays probes. In order to quantify the ALP activity in C2C12 cells induced by BMP2 treatment, 5×10^3^ cells were plated in CellCarrier-96-well™ microplates (Perkin Elmer, Waltham, MA, USA) and cultured in the presence of BMP2±Dipy as described. After 6 days, Hoechst 33342 Nuclear Stain (ENZ-51031-HOE33342, Enzo Life Sciences) was added to the culture medium at a 1:1000 dilution and incubated for 20 min. Cells were then visualized with the NIKON Ti Eclipse microscope; 16 640×490-µm fields for each well and condition were acquired and analyzed by the NIS-Elements AR software to obtain an automated count of the present nuclei. The number of nuclei has been used to normalize the ALP activity measured as follows. After analysis, cells were washed with PBS and incubated with 200 μl of the Alkaline Phosphatase Yellow liquid substrate system (nNPP) (Sigma, Buchs, SG, Switzerland). Reaction was stopped with 60 μl 3 M NaOH and ALP activity measured at 405 nm by Mithras LB940 plate reader (Berthold Technologies).

### Heterotopic ossification *in vivo*

0.05 μg/μl of BMP2 (Peprotech, Rocky Hill, NJ, USA) in 200 μl growth-factor-reduced Matrigel (BD Biosciences) were injected intramuscularly in the quadriceps of C57BL/6 2-month-old mice (11 mice/group). The contralateral muscle was used as internal control and injected with Matrigel only. Both quadriceps were injected with 50 μl cardiotoxin 5 μM (CTX from *Naja mossambica mossambica*, Sigma-Aldrich, Buchs, SG, Switzerland) to increase muscle damage. Animals were anesthetized by inhalation of 2-bromo-2-chloro-1,1,1-trifluoroethane, ≥99% (CAS no. 151-67-7, Sigma-Aldrich, Buchs, SG, Switzerland) before the injection. 10 mg/kg (body weight) dipyridamole was administered daily IP to the treated animals (*n*=11, for Protocol A; *n*=5 for Protocol B, see Fig. S5) in a solution composed of 10% ethanol, 5% 2-pyrolidone, 12-15% propylene glycol, 10% Cremophor ELP, saline to 100% (Wang et al., 2013). Control mice (*n*=11, for Protocol A; *n*=5 for Protocol B, see Fig. S5) received the injection solution without drug. Mice were housed at the San Raffaele Institute SPF animal facility and were kept in pathogen-free conditions. All procedures were in accordance with Italian law and were performed under internal regulations for animal care and handling.

### *In vivo* CT imaging of heterotopic ossification

At day 10 and 21 after BMP injection, *in vivo* micro-computerized tomography (μCT) scans were carried out to assess progression of ossification and any effect on the normal skeletal structure. *In vivo* µCT imaging was performed using the IVIS SpectrumCT Pre-clinical *in vivo* imaging system (Perkin-Elmer, Waltham, MA, USA). CT images were acquired without any contrast medium with the following parameters: x-ray tube voltage=50 kV, tube current=1 mA, x-ray focal spot size=50 μm. The CT images calibrated in Hounsfield unit (HU) were reconstructed with a voxel size of 75 μm^3^. Threshold-based image segmentation were performed to obtain a 3D reconstruction and quantification of the ossification.

The total mineralized volume V=N×voxel size (mm^3^) was quantified using MIPAV (medical imaging processing analysis and visualization) and MATLAB software, where N is the number of voxels corresponding to bone derived from the image segmentation procedure. The bone density quantification was calculated by using the following formula: ∑Ni=1 HUi/V.

### Morphological and histochemical analysis of the heterotopic ossification

At 21 days after BMP injection, muscles were collected and processed for further morphological and histological analyses. BMP/Matrigel-injected and Matrigel-injected quadriceps from treated and control mice were frozen in liquid-nitrogen-cooled isopentane, to allow preparation of 10-μm-thick sections.

Muscle sections were stained with hematoxylin and eosin (H&E) (Sigma-Aldrich, Buchs, SG, Switzerland) or Toluidine Blue (Bio-Optica, Milano, Italy) or Alizarin Red (Sigma-Aldrich, Buchs, SG, Switzerland) according to the manufacturers' instructions. Images were acquired using a Nikon Eclipse E600 microscope (Nikon, Tokyo, Japan). To quantify heterotopic ossification, images of Alizarin-Red-stained sections were subsequently analyzed using the batch mode of the ImageJ vs1.49 macro. The color thresholding algorithm used by this macro is based on an algorithm written by G. Landini (version v1.8) available at http://www.mecourse.com/landinig/software/software.html.

### Immunofluorescence on muscle sections

For immunofluorescence, 8-μm sections from OCT-embedded muscles were fixed with 4% PFA in PBS. They were permeabilized with a 0.2% Triton X-100, 1% BSA solution in PBS for 30 min at RT and then blocked in 10% serum, 1% BSA solution in PBS for 30 min before incubation with the primary antibody p-Smad1/5 (1:800; Cell Signaling, Danvers, MA, USA), after a demasking step in sodium citrate 10 mM pH 6 for 10 min between fixation and blocking steps (2 h). Alexa-Fluor-546-conjugated antibody (1:500; Invitrogen, Thermo Fisher Scientifics, Waltham, MA, USA) was used as second-step reagents. Specimens were counterstained with DAPI (Sigma, Buchs, SG, Switzerland) and analyzed using a Zeiss LSM710 confocal microscope. Images showing double fluorescence were first acquired separately using appropriate filters, then the different layers were merged using Adobe Photoshop CS4.

### Statistical analysis

All luciferase reporter gene assays were performed in triplicate and repeated independently at least twice (2-5 times). Z′ factor was evaluated by using the formula [Z′=1−3×(σ_s_+σ_c_)/|µ_s_−µ_c_|], where σ_s_ and σ_c_ are the s.d. of positive or negative sample and of the solvent (control) and µ_s_ and µ_c_ represent the average. Experiments to evaluate gene expression by RT-qPCR were performed in triplicate from at least two independent RNA extractions. Both the non-parametric Mann–Whitney test (Social Science Statistics) and the unpaired two-tailed Student's *t*-test (GraphPad *t*-test Calculator; http://graphpad.com/quickcalcs/ttest1.cfm) were applied to verify statistical significance of the observed variations. Significant differences were given as *P<0.05, ^#^*P*<0.01 or ^§^*P*<0.001.

## References

[DMM023929C1] Aleman-Muench G. R. and Soldevila G. (2012). When versatility matters: activins/inhibins as key regulators of immunity. *Immunol. Cell Biol.* 90, 137-148. 10.1038/icb.2011.32 21537340

[DMM023929C2] Balakumar P., Nyo Y. H., Renushia R., Raaginey D., Oh A. N., Varatharajan R. and Dhanaraj S. A. (2014). Classical and pleiotropic actions of dipyridamole: not enough light to illuminate the dark tunnel? *Pharmacol. Res.* 87, 144-150. 10.1016/j.phrs.2014.05.008 24861566

[DMM023929C3] Bentzinger C. F., Wang Y. X., Dumont N. A. and Rudnicki M. A. (2013). Cellular dynamics in the muscle satellite cell niche. *EMBO Rep.* 14, 1062-1072. 10.1038/embor.2013.182 24232182 PMC3849491

[DMM023929C4] Bocciardi R., Bordo D., Di Duca M., Di Rocco M. and Ravazzolo R. (2009). Mutational analysis of the ACVR1 gene in Italian patients affected with fibrodysplasia ossificans progressiva: confirmations and advancements. *Eur. J. Hum. Genet.* 17, 311-318. 10.1038/ejhg.2008.178 18830232 PMC2986177

[DMM023929C5] Chaikuad A., Alfano I., Kerr G., Sanvitale C. E., Boergermann J. H., Triffitt J. T., von Delft F., Knapp S., Knaus P. and Bullock A. N. (2012). Structure of the bone morphogenetic protein receptor ALK2 and implications for fibrodysplasia ossificans progressiva. *J. Biol. Chem.* 287, 36990-36998. 10.1074/jbc.M112.365932 22977237 PMC3481300

[DMM023929C6] Chakkalakal S. A., Zhang D., Culbert A. L., Convente M. R., Caron R. J., Wright A. C., Maidment A. D. A., Kaplan F. S. and Shore E. M. (2012). An Acvr1 R206H knock-in mouse has fibrodysplasia ossificans progressiva. *J. Bone Miner. Res.* 27, 1746-1756. 10.1002/jbmr.1637 22508565 PMC3556640

[DMM023929C7] Convente M. R., Yang E., Chakkalakal S. A., Zhang D., Caron R. J., Perrien D. S., Kambayashi T., Kaplan F. S. and Shore E. M. (2015). Targeted ablation of macrophages and mast cells impairs heterotopic ossification in a mouse model of fibrodysplasia ossificans progressiva. *J. Bone Miner. Res.* **30** (Suppl 1), S484. 10.1002/jbmr.3304 PMC7737844 28986986

[DMM023929C8] Costa M. A., Barbosa A., Neto E., Sá-e-Sousa A., Freitas R., Neves J. M., Magalhães-Cardoso T., Ferreirinha F. and Correia-de-Sá P. (2011). On the role of subtype selective adenosine receptor agonists during proliferation and osteogenic differentiation of human primary bone marrow stromal cells. *J. Cell Physiol.* 226, 1353-1366. 10.1002/jcp.22458 20945394

[DMM023929C9] Culbert A. L., Chakkalakal S. A., Theosmy E. G., Brennan T. A., Kaplan F. S. and Shore E. M. (2014). Alk2 regulates early chondrogenic fate in fibrodysplasia ossificans progressiva heterotopic endochondral ossification. *Stem Cells* 32, 1289-1300. 10.1002/stem.1633 24449086 PMC4419363

[DMM023929C10] Cuny G. D., Yu P. B., Laha J. K., Xing X., Liu J.-F., Lai C. S., Deng D. Y., Sachidanandan C., Bloch K. D. and Peterson R. T. (2008). Structure-activity relationship study of bone morphogenetic protein (BMP) signaling inhibitors. *Bioorg. Med. Chem. Lett.* 18, 4388-4392. 10.1016/j.bmcl.2008.06.052 18621530 PMC2570262

[DMM023929C11] de Vos-Koppelaar N. C. M., Kerkhoff H., de Vogel E. M., Zock E. and Dieleman H. G. (2014). The effect of a slower than standard dose escalation scheme for dipyridamole on headaches in secondary prevention therapy of strokes: a randomized, open-label trial (DOSE). *Cerebrovasc. Dis.* 37, 285-289. 10.1159/000360751 24819911

[DMM023929C12] Dresse A., Chevolet C., Delapierre D., Masset H., Weisenberger H., Bozler G. and Heinzel G. (1982). Pharmacokinetics of oral dipyridamole (Persantine) and its effect on platelet adenosine uptake in man. *Eur. J. Clin. Pharmacol.* 23, 229-234. 10.1007/BF00547559 6756935

[DMM023929C13] Funaba M., Ikeda T., Ogawa K., Murakami M. and Abe M. (2003). Role of activin A in murine mast cells: modulation of cell growth, differentiation, and migration. *J. Leukoc. Biol.* 73, 793-801. 10.1189/jlb.0103012 12773512

[DMM023929C14] German D. C., Kredich N. M. and Bjornsson T. D. (1989). Oral dipyridamole increases plasma adenosine levels in human beings. *Clin. Pharmacol. Ther.* 45, 80-84. 10.1038/clpt.1989.12 2910640

[DMM023929C15] Giacopelli F., Cappato S., Tonachini L., Mura M., Di Lascio S., Fornasari D., Ravazzolo R. and Bocciardi R. (2013). Identification and characterization of regulatory elements in the promoter of ACVR1, the gene mutated in Fibrodysplasia Ossificans Progressiva. *Orphanet. J. Rare Dis.* 8, 145 10.1186/1750-1172-8-145 24047559 PMC4015442

[DMM023929C16] Gresele P., Momi S. and Falcinelli E. (2011). Anti-platelet therapy: phosphodiesterase inhibitors. *Br. J. Clin. Pharmacol.* 72, 634-646. 10.1111/j.1365-2125.2011.04034.x 21649691 PMC3195739

[DMM023929C17] Groppe J. C., Wu J., Shore E. M. and Kaplan F. S. (2011). In vitro analyses of the dysregulated R206H ALK2 kinase-FKBP12 interaction associated with heterotopic ossification in FOP. *Cells Tissues Organs* 194, 291-295. 10.1159/000324230 21525719 PMC3178093

[DMM023929C18] Hao J., Ho J. N., Lewis J. A., Karim K. A., Daniels R. N., Gentry P. R., Hopkins C. R., Lindsley C. W. and Hong C. C. (2010). In vivo structure-activity relationship study of dorsomorphin analogues identifies selective VEGF and BMP inhibitors. *ACS Chem. Biol.* 5, 245-253. 10.1021/cb9002865 20020776 PMC2825290

[DMM023929C19] Hatsell S. J., Idone V., Wolken D. M. A., Huang L., Kim H. J., Wang L., Wen X., Nannuru K. C., Jimenez J., Xie L. et al. (2015). ACVR1R206H receptor mutation causes fibrodysplasia ossificans progressiva by imparting responsiveness to activin A. *Sci. Transl. Med.* 7, 303ra137 10.1126/scitranslmed.aac4358 PMC6164166 26333933

[DMM023929C20] He W., Mazumder A., Wilder T. and Cronstein B. N. (2013). Adenosine regulates bone metabolism via A1, A2A, and A2B receptors in bone marrow cells from normal humans and patients with multiple myeloma. *FASEB J.* 27, 3446-3454. 10.1096/fj.13-231233 23682121 PMC3752544

[DMM023929C21] Hino K., Ikeya M., Horigome K., Matsumoto Y., Ebise H., Nishio M., Sekiguchi K., Shibata M., Nagata S., Matsuda S. et al. (2015). Neofunction of ACVR1 in fibrodysplasia ossificans progressiva. *Proc. Natl. Acad. Sci. USA* 112, 15438-15443. 10.1073/pnas.1510540112 26621707 PMC4687587

[DMM023929C22] Kadatz R. (1959). [Pharmacological properties of a new coronary dilator substance 2, 6-bis(diethanolamino)-4,8-dipiperidino-pyrimido[5,4-d]pyrimidine]. *Arzneimittelforschung* 9, 39-45. 13628477

[DMM023929C23] Kan L., Liu Y., McGuire T. L., Berger D. M. P., Awatramani R. B., Dymecki S. M. and Kessler J. A. (2009). Dysregulation of local stem/progenitor cells as a common cellular mechanism for heterotopic ossification. *Stem Cells* 27, 150-156. 10.1634/stemcells.2008-0576 18832590 PMC2752983

[DMM023929C24] Kaplan F. S., Tabas J. A., Gannon F. H., Finkel G., Hahn G. V. and Zasloff M. A. (1993). The histopathology of fibrodysplasia ossificans progressiva. An endochondral process. *J. Bone Joint Surg. Am.* 75, 220-230. 7678595 10.2106/00004623-199302000-00009

[DMM023929C25] Kaplan F. S., Shen Q., Lounev V., Seemann P., Groppe J., Katagiri T., Pignolo R. J. and Shore E. M. (2008). Skeletal metamorphosis in fibrodysplasia ossificans progressiva (FOP). *J. Bone Miner. Metab.* 26, 521-530. 10.1007/s00774-008-0879-8 18979151 PMC3620015

[DMM023929C26] Kaplan F. S., Xu M., Seemann P., Connor J. M., Glaser D. L., Carroll L., Delai P., Fastnacht-Urban E., Forman S. J., Gillessen-Kaesbach G. et al. (2009). Classic and atypical fibrodysplasia ossificans progressiva (FOP) phenotypes are caused by mutations in the bone morphogenetic protein (BMP) type I receptor ACVR1. *Hum. Mutat.* 30, 379-390. 10.1002/humu.20868 19085907 PMC2921861

[DMM023929C27] Kaplan F. S., Pignolo R. J. and Shore E. M. (2013). From mysteries to medicines: drug development for fibrodysplasia ossificans progressiva. *Expert Opin. Orphan. Drugs* 1, 637-649. 10.1517/21678707.2013.825208 24800180 PMC4007356

[DMM023929C28] Kaplan F. S., Pignolo R. J. and Shore E. M. (2016). Granting immunity to FOP and catching heterotopic ossification in the Act. *Semin. Cell. Dev. Biol.* 49, 30-36. 10.1016/j.semcdb.2015.12.013 26706149 PMC4898187

[DMM023929C29] Katagiri T., Yamaguchi A., Komaki M., Abe E., Takahashi N., Ikeda T., Rosen V., Wozney J. M., Fujisawa-Sehara A. and Suda T. (1994). Bone morphogenetic protein-2 converts the differentiation pathway of C2C12 myoblasts into the osteoblast lineage. *J. Cell. Biol.* 127, 1755-1766. 10.1083/jcb.127.6.1755 7798324 PMC2120318

[DMM023929C30] Kim H.-H. and Liao J. K. (2008). Translational therapeutics of dipyridamole. *Arterioscler. Thromb. Vasc. Biol.* 28, S39-S42. 10.1161/ATVBAHA.107.160226 18174451 PMC2615560

[DMM023929C31] Kitoh H., Achiwa M., Kaneko H., Mishima K., Matsushita M., Kadono I., Horowitz J. D., Sallustio B. C., Ohno K. and Ishiguro N. (2013). Perhexiline maleate in the treatment of fibrodysplasia ossificans progressiva: an open-labeled clinical trial. *Orphanet J. Rare Dis.* 8, 163 10.1186/1750-1172-8-163 24131551 PMC4015865

[DMM023929C32] Li Y. Y. and Jones S. J. (2012). Drug repositioning for personalized medicine. *Genome Med.* 4, 27 10.1186/gm326 22494857 PMC3446277

[DMM023929C34] Massaro M., Scoditti E., Carluccio M. A., Pellegrino M., Calabriso N., Storelli C., Martines G. and De Caterina R. (2013). Dipyridamole decreases inflammatory metalloproteinase-9 expression and release by human monocytes. *Thromb. Haemost.* 109, 280-289. 10.1160/TH12-05-0326 23238437

[DMM023929C35] Medici D. and Olsen B. R. (2012). The role of endothelial-mesenchymal transition in heterotopic ossification. *J. Bone Miner. Res.* 27, 1619-1622. 10.1002/jbmr.1691 22806925 PMC3432417

[DMM023929C111] Medici D., Shore E. M., Lounev V. Y., Kaplan F. S., Kalluri R. and Olsen B. R. (2010). Conversion of vascular endothelial cells into multipotent stem-like cells. *Nat. Med.* 16, 1400-1406. 10.1038/nm.2252 21102460 PMC3209716

[DMM023929C36] Mediero A., Wilder T., Perez-Aso M. and Cronstein B. N. (2015). Direct or indirect stimulation of adenosine A2A receptors enhances bone regeneration as well as bone morphogenetic protein-2. *FASEB J.* 29, 1577-1590. 10.1096/fj.14-265066 25573752 PMC4396602

[DMM023929C37] Monteiro R. M., de Sousa Lopes S. M. C., Bialecka M., de Boer S., Zwijsen A. and Mummery C. L. (2008). Real time monitoring of BMP Smads transcriptional activity during mouse development. *Genesis* 46, 335-346. 10.1002/dvg.20402 18615729

[DMM023929C38] Muthyala R. (2011). Orphan/rare drug discovery through drug repositioning. *Drug Discov. Today* 8, 71-76. 10.1016/j.ddstr.2011.10.003

[DMM023929C40] Ogawa K. and Funaba M. (2011). Activin in humoral immune responses. *Vitam. Horm.* 85, 235-253. 10.1016/B978-0-12-385961-7.00012-3 21353884

[DMM023929C42] Oshrine B., Malinin A., Pokov A., Dragan A., Hanley D. and Serebruany V.; Aggrenox Compliance Task Force. (2005). Criticality of pH for accurate fluorometric measurements of dipyridamole levels in biological fluids. *Methods Find Exp. Clin. Pharmacol.* 27, 95-100. 10.1358/mf.2005.27.2.876284 15834462

[DMM023929C44] Rigamonti E., Zordan P., Sciorati C., Rovere-Querini P. and Brunelli S. (2014). Macrophage plasticity in skeletal muscle repair. *Biomed. Res. Int.* 2014, 560629 10.1155/2014/560629 24860823 PMC4016840

[DMM023929C45] Sanvitale C. E., Kerr G., Chaikuad A., Ramel M.-C., Mohedas A. H., Reichert S., Wang Y., Triffitt J. T., Cuny G. D., Yu P. B. et al. (2013). A new class of small molecule inhibitor of BMP signaling. *PLoS ONE* 8, e62721 10.1371/journal.pone.0062721 23646137 PMC3639963

[DMM023929C46] Sardana D., Zhu C., Zhang M., Gudivada R. C., Yang L. and Jegga A. G. (2011). Drug repositioning for orphan diseases. *Brief Bioinform.* 12, 346-356. 10.1093/bib/bbr021 21504985

[DMM023929C47] Seeger P., Bosisio D., Parolini S., Badolato R., Gismondi A., Santoni A. and Sozzani S. (2014). Activin A as a mediator of NK-dendritic cell functional interactions. *J. Immunol.* 192, 1241-1248. 10.4049/jimmunol.1301487 24395917

[DMM023929C48] Serebruany V., Sabaeva E., Booze C., Atar O. D., Eisert C. and Hanley D. (2009). Aggrenox Compliance Task Force. Distribution of dipyridamole in blood components among post-stroke patients treated with extended release formulation. *Thromb. Haemost.* 2, 538-543. 10.1160/TH09-03-0158 19718475

[DMM023929C49] Shameer K., Readhead B. and Dudley J. T. (2015). Computational and experimental advances in drug repositioning for accelerated therapeutic stratification. *Curr. Top. Med. Chem.* 15, 5-20. 10.2174/1568026615666150112103510 25579574

[DMM023929C50] Sheng N., Xie Z., Wang C., Bai G., Zhang K., Zhu Q., Song J., Guillemot F., Chen Y.-G., Lin A. et al. (2010). Retinoic acid regulates bone morphogenic protein signal duration by promoting the degradation of phosphorylated Smad1. *Proc. Natl. Acad. Sci. USA* 107, 18886-18891. 10.1073/pnas.1009244107 20956305 PMC2973900

[DMM023929C51] Shimono K., Tung W.-E., Macolino C., Chi A. H.-T., Didizian J. H., Mundy C., Chandraratna R. A., Mishina Y., Enomoto-Iwamoto M., Pacifici M. et al. (2011). Potent inhibition of heterotopic ossification by nuclear retinoic acid receptor- γ agonists. *Nat. Med.* 17, 454-460. 10.1038/nm.2334 21460849 PMC3073031

[DMM023929C52] Shore E. M. (2012). Fibrodysplasia ossificans progressiva: a human genetic disorder of extraskeletal bone formation, or--how does one tissue become another? *Wiley Interdiscip. Rev. Dev. Biol.* 1, 153-165. 10.1002/wdev.9 22408652 PMC3297114

[DMM023929C53] Shore E. M., Feldman G. J., Xu M. and Kaplan F. S. (2005). The genetics of fibrodysplasia ossificans progressiva. *Clin. Rev. Bone Miner. Metab.* 3, 201-204. 10.1385/BMM:3:3-4:201

[DMM023929C54] Shore E. M., Xu M., Feldman G. J., Fenstermacher D. A., Cho T. J., Choi I. H., Connor J. M., Delai P., Glaser D. L., LeMerrer M. et al. (2006). A recurrent mutation in the BMP type I receptor ACVR1 causes inherited and sporadic fibrodysplasia ossificans progressiva. *Nat. Genet.* 38, 525-527. 10.1038/ng1783 16642017

[DMM023929C55] Song G.-A., Kim H.-J., Woo K.-M., Baek J.-H., Kim G.-S., Choi J.-Y. and Ryoo H.-M. (2010). Molecular consequences of the ACVR1(R206H) mutation of fibrodysplasia ossificans progressiva. *J. Biol. Chem.* 285, 22542-22553. 10.1074/jbc.M109.094557 20463014 PMC2903413

[DMM023929C56] Tare R. S., Howard D., Pound J. C., Roach H. I. and Oreffo R. O. C. (2005). Tissue engineering strategies for cartilage generation—micromass and three dimensional cultures using human chondrocytes and a continuous cell line. *Biochem. Biophys. Res. Commun.* 333, 609-621. 10.1016/j.bbrc.2005.05.117 15946652

[DMM023929C57] van Dinther M., Visser N., de Gorter D. J., Doorn J., Goumans M. J., de Boer J. and ten Dijke P. (2010). ALK2 R206H mutation linked to fibrodysplasia ossificans progressiva confers constitutive activity to the BMP type I receptor and sensitizes mesenchymal cells to BMP-induced osteoblast differentiation and bone formation. *J. Bone Miner. Res.* 25, 1208-1215. 10.1359/jbmr.091110 19929436

[DMM023929C58] Visser F., Baldwin S. A., Isaac R. E., Young J. D. and Cass C. E. (2005). Identification and mutational analysis of amino acid residues involved in dipyridamole interactions with human and Caenorhabditis elegans equilibrative nucleoside transporters. *J. Biol. Chem.* 280, 11025-11034. 10.1074/jbc.M410348200 15649894

[DMM023929C59] Wang C., Schwab L. P., Fan M., Seagroves T. N. and Buolamwini J. K. (2013). Chemoprevention activity of dipyridamole in the MMTV-PyMT transgenic mouse model of breast cancer. *Cancer Prev. Res.* 6, 437-447. 10.1158/1940-6207.CAPR-12-0345 PMC3829204 23447563

[DMM023929C60] Yamamoto R., Matsushita M., Kitoh H., Masuda A., Ito M., Katagiri T., Kawai T., Ishiguro N. and Ohno K. (2013). Clinically applicable antianginal agents suppress osteoblastic transformation of myogenic cells and heterotopic ossifications in mice. *J. Bone Miner. Metab.* 31, 26-33. 10.1007/s00774-012-0380-2 23011467

[DMM023929C61] Yip S. and Benavente O. (2011). Antiplatelet agents for stroke prevention. *Neurotherapeutics* 8, 475-487. 10.1007/s13311-011-0060-2 21761240 PMC3250274

[DMM023929C62] Yu P. B., Deng D. Y., Lai C. S., Hong C. C., Cuny G. D., Bouxsein M. L., Hong D. W., McManus P. M., Katagiri T., Sachidanandan C. et al. (2008a). BMP type I receptor inhibition reduces heterotopic ossification. *Nat. Med.* 14, 1363-1369. 10.1038/nm.1888 19029982 PMC2846458

[DMM023929C63] Yu P. B., Hong C. C., Sachidanandan C., Babitt J. L., Deng D. Y., Hoyng S. A., Lin H. Y., Bloch K. D. and Peterson R. T. (2008b). Dorsomorphin inhibits BMP signals required for embryogenesis and iron metabolism. *Nat. Chem. Biol.* 4, 33-41. 10.1038/nchembio.2007.54 18026094 PMC2727650

[DMM023929C64] Zhang J.-H., Chung T. D. Y. and Oldenburg K. R. (1999). A Simple Statistical Parameter for Use in Evaluation and Validation of High Throughput Screening Assays. *J. Biomol. Screen* 4, 67-73. 10.1177/108705719900400206 10838414

[DMM023929C65] Zordan P., Rigamonti E., Freudenberg K., Conti V., Azzoni E., Rovere-Querini P. and Brunelli S. (2014). Macrophages commit postnatal endothelium-derived progenitors to angiogenesis and restrict endothelial to mesenchymal transition during muscle regeneration. *Cell Death Dis.* 5, e1031 10.1038/cddis.2013.558 24481445 PMC4040684

