# Correction: High-throughput screening for modulators of *ACVR1* transcription: discovery of potential therapeutics for fibrodysplasia ossificans progressiva

**DOI:** 10.1242/dmm.027573

**Published:** 2016-09-01

**Authors:** Serena Cappato, Laura Tonachini, Francesca Giacopelli, Mario Tirone, Luis J. V. Galietta, Martina Sormani, Anna Giovenzana, Antonello E. Spinelli, Barbara Canciani, Silvia Brunelli, Roberto Ravazzolo, Renata Bocciardi

There was an error concerning Fig. 4 in *Dis. Model. Mech.* **9**, 685-696.

The wrong figure was used for Fig. 4. The correct figure is shown in this Correction with its legend. The PDF and full-text version of this article have been corrected.

**Fig. 4.**
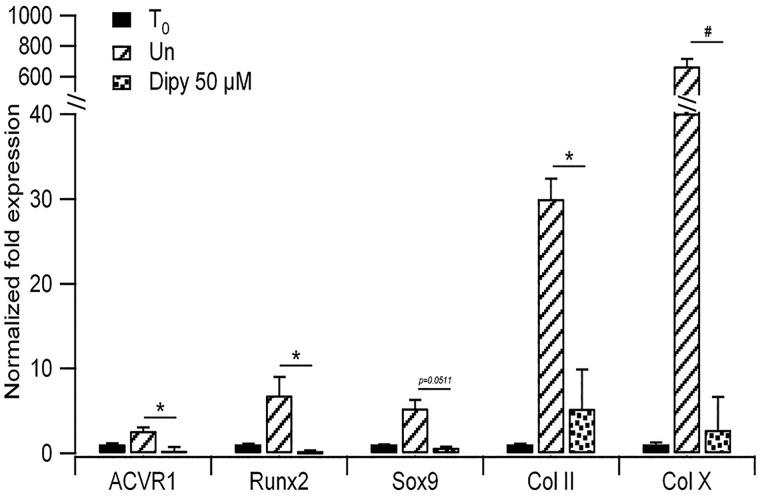
**Effect of Dipy on the expression of chondrogenic markers.** RT-qPCR on RNA extracted from ATDC5 cells cultured as alginate spheres for 14 days in differentiation medium. Bars show mean and s.d. of three independent experiments. Expression levels were normalized on *GAPDH* and *18S* and compared to that of cells at T_0_ (cells harvested at the beginning of the differentiation protocol). Un, untreated cells. ns, non-significant; *P<0.05, ^§^*P*<0.001.

The authors and DMM office apologise to the readers for this error.

